# Successful Robot-Assisted Simple Prostatectomy Years After a Prostate Artery Embolization: A Case Report and Review of the Literature

**DOI:** 10.7759/cureus.54575

**Published:** 2024-02-20

**Authors:** Kale Moreland, Gabrielle R Yankelevich, Paul Womble

**Affiliations:** 1 Department of Urology, Kansas City University College of Osteopathic Medicine, Kansas City, USA; 2 Department of Urology, Medical University of South Carolina, Charleston, USA

**Keywords:** bph management, benign prostatic hyperplasia (bph), hematuria, simple prostatectomy, prostate artery embolization

## Abstract

Simple prostatectomy (SP) can be utilized for patients with large prostates with lower urinary tract symptoms. Prostate artery embolization (PAE) does not have robust clinical evidence to support its use in treating urinary symptoms; however, it is an effective treatment for refractory hematuria from a prostatic source. There have been limited papers regarding preoperative PAE prior to SP. However, there are no papers regarding the feasibility of delayed SP after PAE. We present, to our knowledge, the first paper demonstrating a successful robot-assisted SP years after a PAE in a patient with a 380g prostate with recurrent refractory gross hematuria.

## Introduction

Benign prostatic hyperplasia (BPH) is a condition that affects men at an increasing rate as they age, with an 8-60% prevalence by 90 years old [1]. The American Urological Association’s (AUA) guidelines state that patients who have lower urinary tract symptoms (LUTS) with large prostates (> 80 grams) can undergo laser enucleation of the prostate or simple prostatectomy (SP) [2]. The guidelines recommend against prostate artery embolization (PAE) for the treatment of LUTS outside of clinical trials as current literature is sparse and provides mixed results when comparing PAE to transurethral resection of the prostate (TURP) [2]. However, PAE has been utilized for refractory hematuria from a prostatic source with success rates between 80 and 100% at one year [3,4]. Moreover, there is sparse literature regarding performing an SP after PAE. Of the literature that exists (three papers), the average prostate volume prior to embolization was ~100-130g and the average prostate volume resected was ~60g [5-7]. Lastly, the limited literature discusses preoperative PAE prior to SP as a means of decreasing intraoperative bleeding, rather than the feasibility of performing SP months to years after PAE has been performed. 

We present, to our knowledge, the first paper for a successful robot-assisted simple prostatectomy (RASP) years after a PAE. Additionally, we highlight the fact that our patient had a 380g prostate-three times larger than the average prostates reported in prior case series. 

## Case presentation

This is an 81-year-old male with a past medical history of hyperlipidemia, chronic kidney disease (eGFR 54), legal blindness, and prostatomegaly who presented with gross hematuria with clot retention. The patient previously had four negative prostate biopsies from 1999 to 2007 for elevated PSA in the setting of prostatomegaly. When he presented to us in 2016 with recurrent hematuria with clot retention, he was recommended to undergo an SP or PAE due to his prostate size being 280g. Despite extensive counseling, he opted against these recommendations and underwent TURP for hemostasis. Final pathology identified Gleason 3+3 in 6/181 TURP chips with a total volume of 31g resected. Given the diagnosis of low-risk prostate cancer, he opted for active surveillance. In 2019, he returned with gross hematuria, and at that time, he elected to proceed with PAE over SP. 

**Figure 1:**
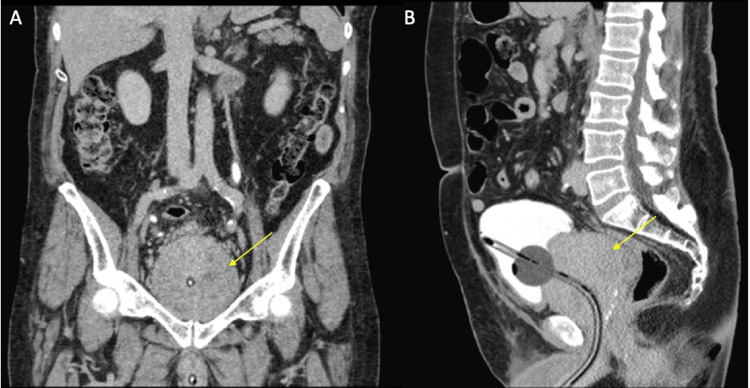
Coronal (A) and sagittal views (B) of 380g prostate (yellow arrow) with the Foley catheter in place (with 40cc in balloon for reference).

In 2023, he again presented to the emergency room with gross hematuria. He was re-imaged which demonstrated a prostate size of approximately 380g (Figure 1). His PSA several months prior was 9.4 ng/ml (corrected for finasteride to 18.8 ng/ml), with a stable PSA density of 0.05. A three-way catheter was placed and irrigated, and he was started on continuous bladder irrigation. He failed several clamp trials and was recommended to undergo RASP, which after several days he was amenable to. Given low-risk disease, stable PSA, and stable PSA density, he was recommended to undergo RASP rather than radical prostatectomy. 

An intravesical approach was taken for the RASP and then the posterior bladder neck was incised to the level of the adenoma. The adenoma was attempted to be dissected circumferentially, but given its size, portions of the adenoma were transected and set aside to allow for visualization. Judicious use of electrocautery and a running 3-0 V-lock suture were utilized for hemostasis within the adenoma defect. The posterior bladder neck was advanced within the prostate and sutured at the 5 and 7 o’clock positions. Floseal was placed in the fossa, a 22 French three-way catheter was placed with 70mL in the balloon, and the catheter was placed on traction. The cystostomy was closed in a water-tight fashion in two layers using a 2-0 V-lock. A drain was placed within the abdomen and once the robot was undocked, the catheter was placed on CBI. 

Postoperative day one, the patient required two units of packed red blood cells (pRBC) for anemia. He was discharged on postoperative day eleven. His course was complicated by anemia requiring a total of five units of pRBC, postoperative ileus, and leukocytosis with positive E. coli urine culture but was able to be transitioned to oral antibiotics. The Foley catheter was removed postoperative day 9 and he was able to empty completely. 

The patient was seen two weeks postoperatively to discuss the pathology which was 138g of benign prostate tissue. He obtained a PSA at three months postoperatively which was 1.48 (off finasteride). At this visit, he did report non-bothersome urgency and occasional urgency incontinence but overall was satisfied with his symptoms. He was most recently seen at seven months postoperatively, where he reported a strong stream at home and had a PVR in the office of 16cc. His IPSS score was 3, consistent with mild symptoms. 

## Discussion

SP has been studied extensively as a treatment for BPH with large prostates, and PAE has been shown in small studies to be an effective treatment for refractory gross hematuria; however, limited analyses have been conducted on SP following PAE. While our case study is limited by its follow-up time, it demonstrates the successful management of a very large prostate using SP years after the patient underwent PAE. 

A retrospective analysis by Kam et al. analyzed 11 patients who had undergone SP following PAE [5]. The mean prostate size in this analysis was 129.7g, with the upper limit reaching 194.8 mL. All patients showed improvement regarding symptom scores, PSA level, maximum flow rate, and prostatic volume. An additional analysis of 18 patients by Sare et al. specifically examined perioperative blood loss with SP following PAE [6]. The mean prostate size in this study was 128.8 g, with the upper limit reaching 162.4 g. A statistically significant reduction in perioperative blood loss was shown with SP following PAE compared to SP alone. Lastly, Shin et al. performed a retrospective analysis on performing a preoperative PAE followed by RASP or holmium-laser enucleation (HoLEP) [7]. They included 10 patients who underwent PAE followed by HoLEP (six patients) or RASP (four patients) and compared them to a control group without preoperative PAE. The mean prostate volume was 106.5g. They found decreased blood loss and decreased length of hospitalization for the preoperative PAE group. Though these retrospective analyses provide promising evidence for the safety and efficacy of SP following PAE, prospective studies are needed to validate their findings. 

To our knowledge, this is the first report of a successful SP following PAE for a prostate of this size. Moreover, there have been no papers published on the feasibility of performing an RASP years after a PAE. Future randomized control trials to investigate preoperative PAE prior to a RASP as well as retrospective studies on patients who underwent RASP after PAE are prudent. 

## Conclusions

In our experience, RASP is feasible after PAE, even in a delayed fashion. Our hope in providing this information is that it will give clinicians and researchers an initial evidence point for the safety and efficacy of SP following PAE for exceedingly large prostates and it serves as a catalyst for further exploration into the navigation of treatment options for patients with prostates of this size. As the incidence of BPH increases, clinicians will need to continually push the status quo in order to optimize outcomes in this ever-evolving condition. 
